# Supplementary material for: Virulence and Genetic Diversity of Puccinia spp., Causal Agents of Rust on Switchgrass (Panicum virgatum L.) in the USA
Source: Pathogens. 2025 Feb 14;14(2):194. doi: 10.3390/pathogens14020194 (PMC11858125; doi:10.3390/pathogens14020194)
Supplement: Supplementary file 1 [file pathogens-14-00194-s001.zip › Supplementary figures May25-Dec23-Dec28-Feb13.pdf]

SUPPLEMENTARY FIGURES

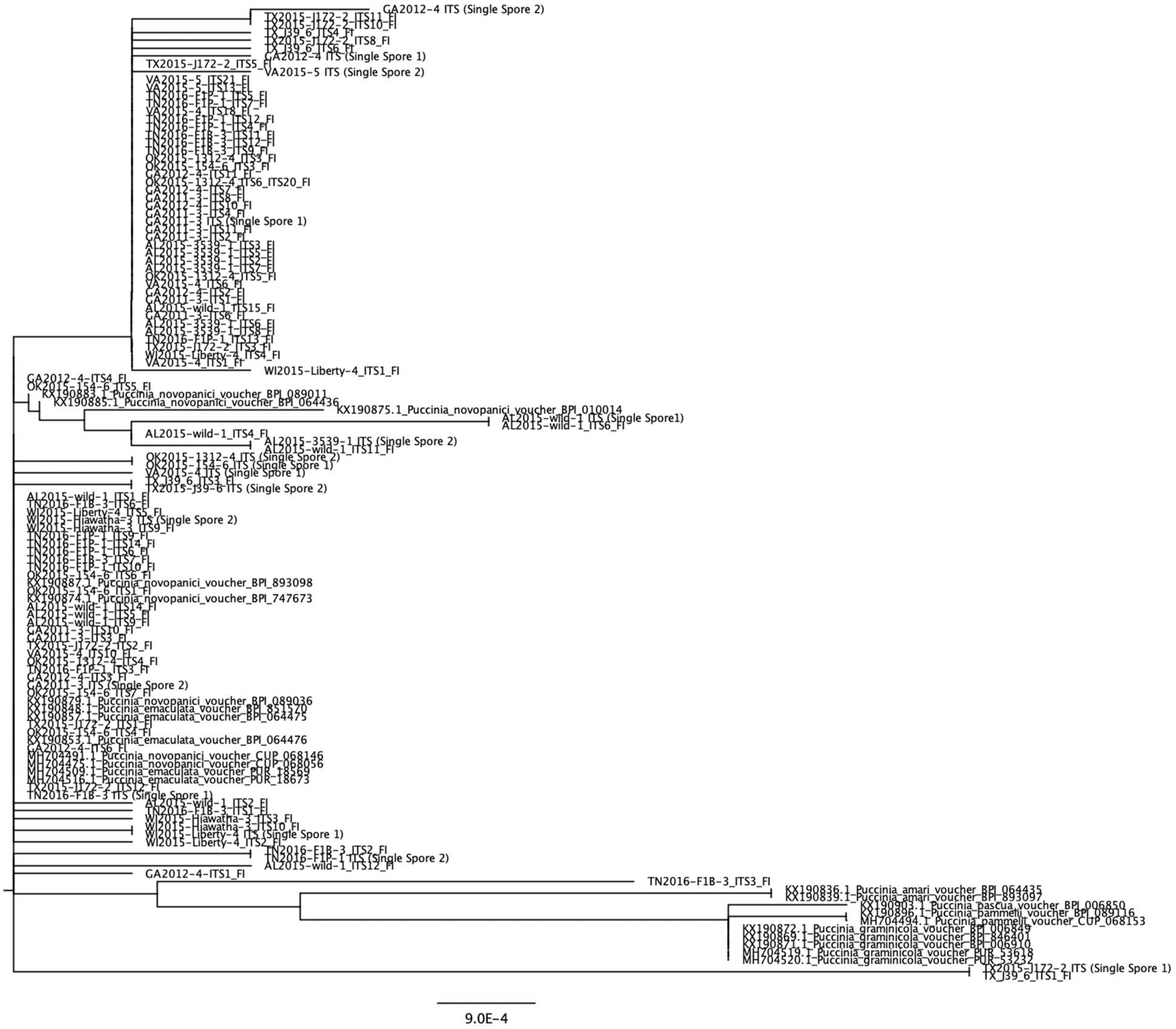

Figure S1. Maximum likelihood tree based on 928bp of ITS sequenced region, showing the phylogenetic relationship between *Puccinia* spp. haplotypes from switchgrass, performed under Mega X v10.1.7, using the Tamura-Nei model and 1000 bootstrap replications. Four clones of each of the 14 single-spore isolates, 4-10 clones of each of the 14 field samples, and 23 published *Puccinia* reference sequenced from six species were used.

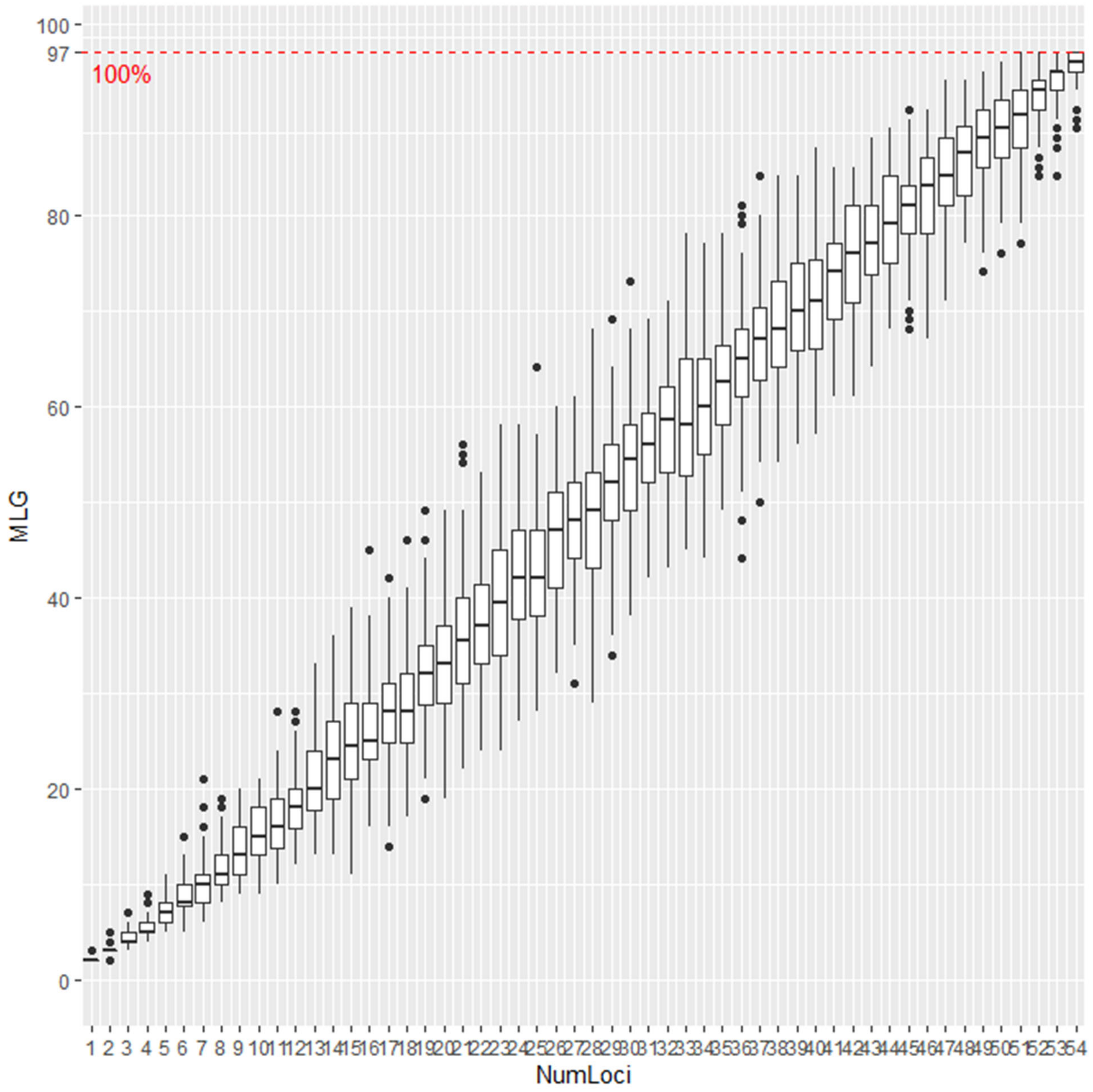

Figure S2. Genotype accumulation curve generated under R software v3.6.1 for amplicon sequences generated for the *EF-1α* gene from *Puccinia* spp. from 14 field isolates from switchgrass sampled in seven U.S. states, showing the informativeness of the genetic diversity analysis.

(A)

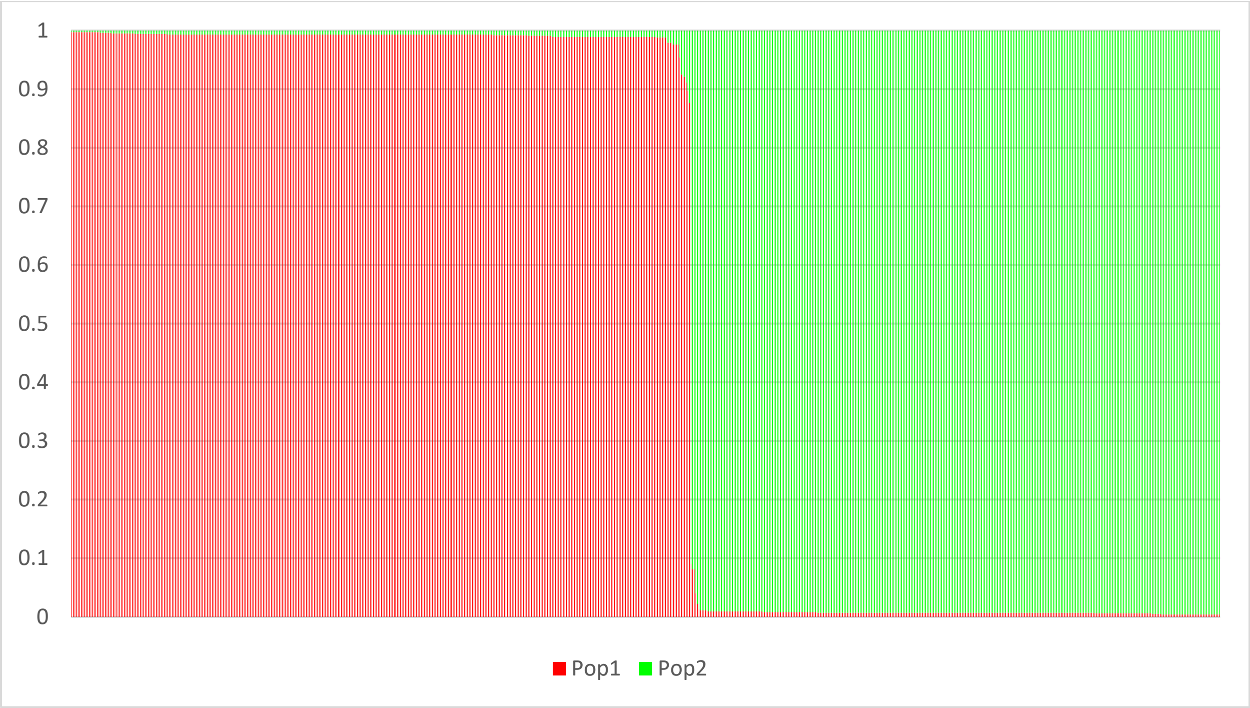
1
0.9
0.8
0.7
0.6
0.5
0.4
0.3
0.2
0.1
0
Pop1
Pop2

(B)

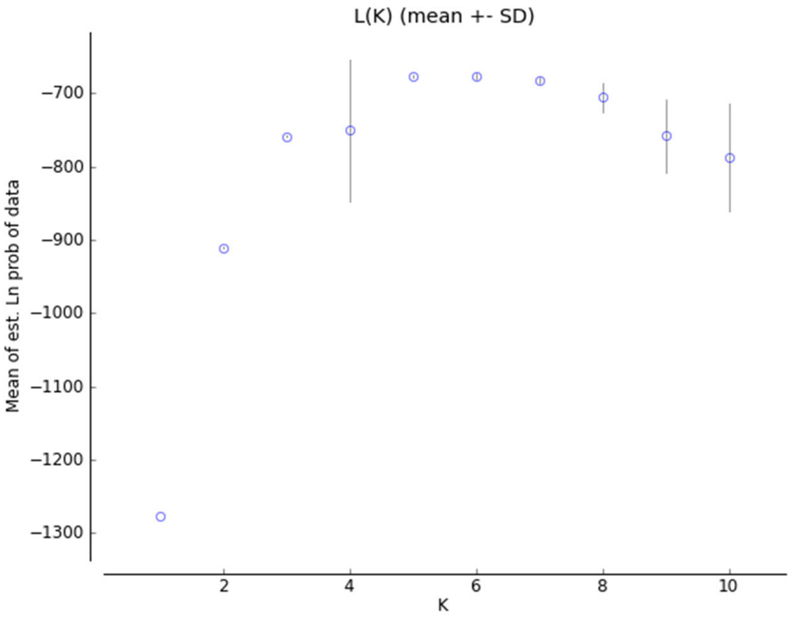
L(K) (mean +- SD)
Mean of est. Ln prob of data
−700
−800
−900
−1000
−1100
−1200
−1300
2
4
6
8
10
K

(C)

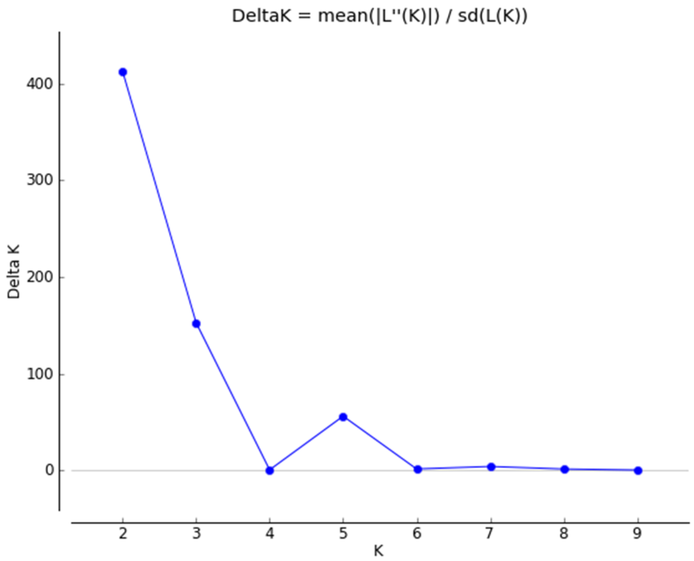

Figure S3. STRUCTURE output for 720 rust switchgrass haplotypes based on 39 SNPs in the *EF-1α* gene: (A) Haplotype's estimated membership probability to each genetic cluster at K=2 (Pop1 and Pop2 are in red and green, respectively), where haplotypes are represented by a thin vertical line and were grouped by genetic cluster; (B) Log probability of data as a function of K; (C) Evanno et al. (2005) plot for detecting the number of K clusters that best fit the data. STRUCTURE was run for K ranging from 1 to 10, and 10 repetitions were performed with 100,000 burn-ins and 100,000 runs. K = 2 clusters were retained as the most likely number of genetic clusters in the rust switchgrass population analyzed.

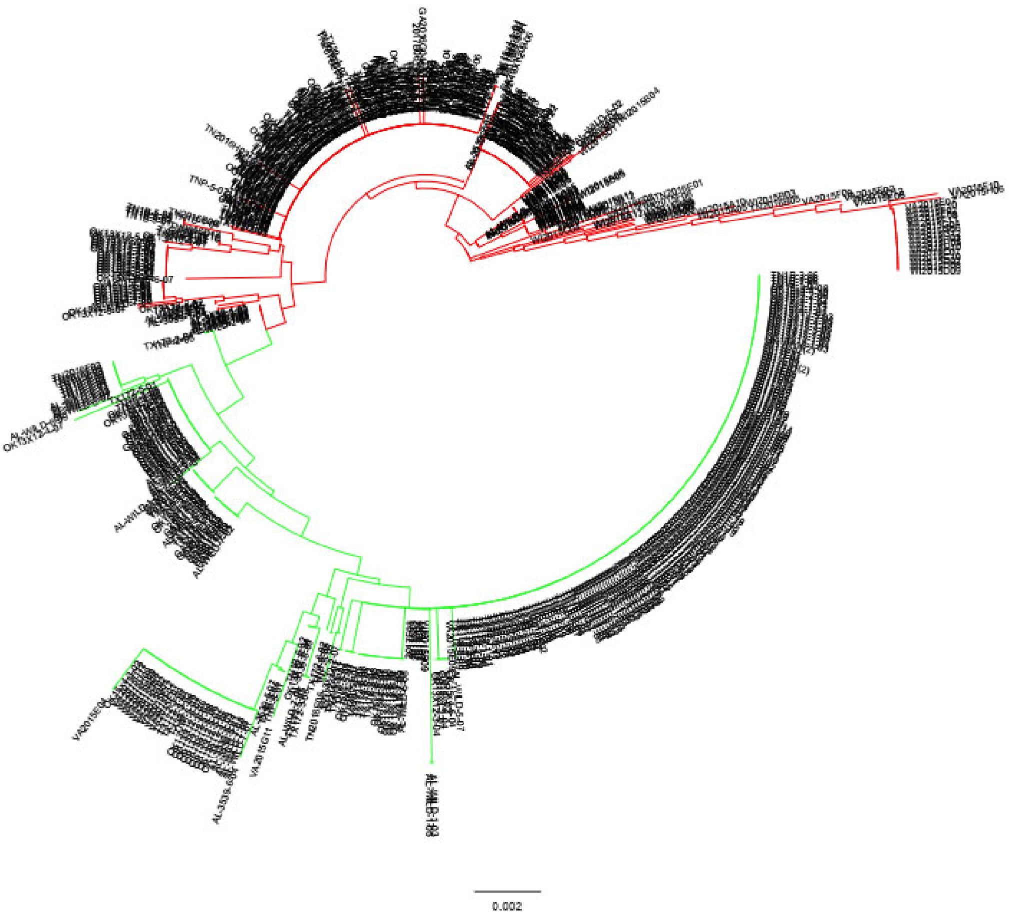

Figure S4. Neighbor-joining tree showing evolutionary relationships between 720 *Puccinia* spp. haplotypes based on 39 SNPs in the *EF-1α* gene. The red and green branches indicate Pop1 and Pop2 genetic clusters, respectively.
